# Supplementary material for: COZOID: contact zone identifier for visual analysis of protein-protein interactions
Source: BMC Bioinformatics. 2018 Apr 6;19:125. doi: 10.1186/s12859-018-2113-6 (PMC5889581; doi:10.1186/s12859-018-2113-6)
Supplement: Supplementary file 2 — Software build. Executable binary file of the software tool. (ZIP 118,784 kb) [file 12859_2018_2113_MOESM2_ESM.zip › cozoid/platform/docs/testng-6.5.1-javadoc.zip!/org/testng/IInvokedMethodListener2.html]

IInvokedMethodListener2 (TestNG)


---


|  |  |  |
| --- | --- | --- |
| **Overview** \| **Package** \| **Class** \| **Tree** \| **Deprecated** \| **Index** \| **Help** | |  |
| **PREV CLASS**   **NEXT CLASS** | **FRAMES**    **NO FRAMES**     **All Classes** |  |
| SUMMARY: NESTED \| FIELD \| CONSTR \| METHOD | DETAIL: FIELD \| CONSTR \| METHOD |  |


---


## org.testng Interface IInvokedMethodListener2

**All Superinterfaces:**: IInvokedMethodListener, ITestNGListener

---

``` public interface IInvokedMethodListener2 extends IInvokedMethodListener ```

Implement this interface if you need a handle to `ITestContext`.

**Author:**
:   karthik.krishnan@gmail.com (Karthik Krishnan)

---

| **Method Summary** | |
| --- | --- |
| `void` | `afterInvocation(IInvokedMethod method, ITestResult testResult, ITestContext context)`             To be implemented if the method needs a handle to contextual information. |
| `void` | `beforeInvocation(IInvokedMethod method, ITestResult testResult, ITestContext context)`             To be implemented if the method needs a handle to contextual information. |

**Methods inherited from interface org.testng.IInvokedMethodListener**

`afterInvocation, beforeInvocation`

**Method Detail**

### beforeInvocation

```
void beforeInvocation(IInvokedMethod method,
                      ITestResult testResult,
                      ITestContext context)
```

:   To be implemented if the method needs a handle to contextual information.

---


### afterInvocation

```
void afterInvocation(IInvokedMethod method,
                     ITestResult testResult,
                     ITestContext context)
```

:   To be implemented if the method needs a handle to contextual information.


---


|  |  |  |
| --- | --- | --- |
| **Overview** \| **Package** \| **Class** \| **Tree** \| **Deprecated** \| **Index** \| **Help** | |  |
| **PREV CLASS**   **NEXT CLASS** | **FRAMES**    **NO FRAMES**     **All Classes** |  |
| SUMMARY: NESTED \| FIELD \| CONSTR \| METHOD | DETAIL: FIELD \| CONSTR \| METHOD |  |


---
